# Platelet-Rich Fibrin Treatment Evaluation in Patients with Medication-Related Osteonecrosis of the Jaw and Osteoradionecrosis

**DOI:** 10.3390/jcm13123473

**Published:** 2024-06-14

**Authors:** Rareș Călin Roman, Mădălina Anca Moldovan, Loredana Sabrina Pop, Sergiu Megieșan, Cosmin Ioan Faur

**Affiliations:** 1 Department of Oral and Maxillofacial Surgery and Implantology, Iuliu Hatieganu University of Medicine and Pharmacy Cluj-Napoca, 400006 Cluj-Napoca, Romania; raresroman@yahoo.com; 2 Faculty of Dental Medicine, Iuliu Hatieganu University of Medicine and Pharmacy Cluj-Napoca, 400006 Cluj-Napoca, Romania; sabrinapop95@gmail.com; 3 Department of Mathematics, Imperial College London Alumni, London SW7 2AZ, UK; serg.flor99@gmail.com; 4 Department of Oral Radiology, Iuliu Hatieganu University of Medicine and Pharmacy Cluj-Napoca, 400006 Cluj-Napoca, Romania; cosmin.faur@yahoo.com

**Keywords:** medication-related osteonecrosis of the jaw, osteoradionecrosis, platelet-rich fibrin, iodoform gauze, primary suture, recurrence, bisphosphonates, radiotherapy

## Abstract

**Background.** Medication-related osteonecrosis of the jaw (MRONJ) and osteoradionecrosis (ORN) are associated with severe disability and continuous pain, both of which are very difficult to control. This study aims to evaluate the outcome of platelet-rich fibrin (PRF) treatment compared to iodoform gauze packing and the primary suture of oral mucosa in patients with both MRONJ and ORN. **Methods.** Patients suffering from MRONJ and ORN who were treated in the Oral and Maxillofacial Surgery Clinic of Cluj-Napoca in the last 10 years were selected for this study from the hospital database. **Results.** PRF treatment proved to be a reliable method to help heal the necrotic bone sites. High-ASA risk patients and immunosuppressed patients are more prone to recurrence and persistent signs and symptoms. Intravenous bisphosphonates produce more intense symptomatology compared to oral administration. The posterior mandible is more difficult to treat compared to other sites. **Conclusions.** The quality of life of MRONJ and ORN patients may be improved by a protocol that reduces pain and hospitalization.

## 1. Introduction

Bisphosphonates (BPs) and monoclonal antibodies are antiresorptive and antiangiogenic therapies used to modulate bone metabolism in different pathologies, such as osteoporosis or malignant lesions [1]. While low doses are required in essential bone disease, such as osteoporosis, higher doses are needed in the treatment of malignant tumors or bone metastatic disease [2]. BPs affect osteoclastic activity and bone remodeling, while monoclonal antibodies prevent the formation, differentiation, and function of osteoclasts [3]. Even though bisphosphonates and monoclonal antibodies are used for the same conditions, BPs are the first-line treatment and are preferred in patients with a higher fracture risk, while monoclonal antibodies (Denosumab) are used for patients who cannot tolerate BPs or have renal impairments [4,5,6]. The main disadvantage of these therapies is the occurrence of medication-related osteonecrosis of the jaw (MRONJ) [1]. The American Association of Oral and Maxillofacial Surgeons (AAOMS) defined MRONJ using three important criteria that needed to be checked: exposed necrotic bone that persisted for more than eight weeks, antiresorptive or antiangiogenic therapy in current or previous administration, and no history of radiation to the jaw or malignant jaw lesions [7]. The major risk factor when it comes to MRONJ occurrence is dentoalveolar surgery, with patients with associated comorbidities being prone to developing complications [7,8]. 

Osteoradionecrosis (ORN) is a secondary effect of head and neck radiotherapy [9]. The definition of ORN relates to the exposed necrotic bone that persists for longer than three months, with the absence of a local tumor [10]. The three main phenomena that appear after radiotherapy and facilitate the occurrence of ORN are hypovascularization, hypocellularity, and tissue hypoxemia, leading to a weaker ability to heal [9]. As well as MRONJ, ORN occurrence is associated with systemic factors, alcohol, cigarette consumption, and dentoalveolar surgery or trauma [11]. 

The current therapies established for both MRONJ and ORN are conservative (antibiotics and antiseptics) and surgical (debridement and curettage resections) [12]. To potentially aid healing, platelet-rich fibrin (PRF) has been used as an adjuvant therapy [11]. PRF is a second-generation autologous platelet concentrate (APC) [12]. To prepare PRF, the patient’s blood is collected and separated by centrifugation [13]. Due to this process, a fibrin clot with platelets, leukocytes, immune cytokines, and the circulating stem cell is gained [13]. Growth factors released by platelets stimulate regeneration and wound healing [14]. 

MRONJ and ORN therapies are still debated in the literature. However, the epidemiology and treatment protocol of MRONJ and ORN have not been reported in Romania.

This study aims to evaluate the treatment outcomes of PRF treatment compared to iodoform gauze packing and the primary suture of oral mucosa, and not only to highlight therapeutic results according to individual risk factors, but also to compare the results of these therapeutic methods in terms of healing and the risk of recurrence in patients with both MRONJ and ORN.

## 2. Materials and Methods

### 2.1. Patient Selection

Patients admitted to the Oral and Maxillofacial Surgery Department of Cluj-Napoca County Hospital in the last 10 years with a clinical and radiological diagnosis of MRONJ and ORN were included in this study. We excluded patients who presented abscesses at the admission time and who presented an active malignant tumor in the adjacent regions.

The patient’s clinical and demographic data were retrospectively obtained from the Cluj-Napoca County Emergency Clinical Hospital’s database (Atlas Med, Gama IT, Sibiu, Romania). The retrieved details were personal data, information about pathology (tumor/osteoporosis) and medication, medical history and American Society of Anesthesiologists (ASA) risk, and a questionnaire were applied to patients to evaluate the signs and symptoms before and after treatment. However, not all patients filled out the questionnaire [15]. All the patients were examined by Cone Beam Computer Tomography prior to the treatment. The patients’ bone necrosis was classified according to localization into the anterior and posterior mandible and maxilla, due to the different vascularization pattern. Therefore, the bone anterior to the canine was considered the anterior region, while the bone located distal to the canine was considered the posterior region.

### 2.2. Treatment and Follow-Up Protocol

All the patients were surgically treated under local anesthesia or sedation by necrotomy (sequestrectomy) and planning of the affected bone, with no or minimal exposure of the jawbone. A sample of bone was sent for microbiologic examination and the antibiotic treatment was adjusted accordingly. Also, another sample was harvested from the cancer site to exclude any local recurrence or metastatic disease. The wound was cleansed with antiseptic solutions and further protected by PRF or iodoform gauze packing or was primarily sutured. Patients were given postoperative instructions and a medical prescription, which included antibiotics and nonsteroidal anti-inflammatory drugs. Also, a follow-up program was set, with visits at 7 days, 14 days, 1 month, 3 months, and 6 months for all patients. Clinical aspects were analyzed each time, while radiographic aspects were analyzed using OPT at 3 months and CT scans at 6 months [16].

The classification of MRONJ was completed using the AAOMS staging system. Stage 0 was considered when patients had no clinical evidence or non-specific clinical evidence and non-specific radiographic findings. Stage 1 presented exposed and necrotic bone, or fistulae that probes to bone, associated with an asymptomatic patient, with no evidence of infection. Stage 2 shows exposed and necrotic bone, or fistulae that probes to the bone, associated with infection, pain, and erythema in the region of the exposed bone, with or without purulent drainage. Stage 3 shows exposed and necrotic bone, or fistulae that probes to bone, associated with pain, infection, and one or more of the following observations: exposed necrotic bone extending beyond the alveolar bone region, resulting in pathological fracture; extraoral fistula, oro-antral, or oro-nasal communication; and/or lytic changes extending to the lower border of the mandible or sinus floor [17]. 

For ORN, Epstein et al.’s classification was used, which grades bone necrosis by progression. Type I shows resolved and healed bone, with (a) or without (b) pathologic fracture. Type II shows chronic persistent (nonprogressive) necrosis, with (a) or without (b) pathologic fracture. Type III shows active progressive necrosis, with (a) or without (b) pathologic fracture [17].

The PRF preparation was performed using the A-PRF protocol. A ten-millilitre tube was used to harvest blood from a venous puncture. The blood was centrifuged at 1300 rotations per minute for 8 min at room temperature using the XC 3000 Spinplus Centrifuge (China) and 10 mL A-PRF tubes, as this distribution method ensures a more even platelet distribution [18]. The PRF clot was further collected by separating the white part from the red part, and was then inserted into the defect, finally being stabilized by sutures.

The outcome was described as healed if there was a complete resolution of symptoms and an absence of signs. The outcome was categorized as recurrence if there were persistent or aggravated signs and symptoms, or if they reappeared over time.

### 2.3. Statistical Analysis

Python 3.10 (Jupyter Notebook, New York, NY, USA) Software was used for the statistical analysis with the following techniques: trend visualization (Linear Regression), Pie Charts, Bar Plots, Correlation Matrices, Distribution Matrices, and statistical tests (Kolmogorov–Smirnov, Kruskal–Wallis, and chi-squared tests). The statistical significance was set as *p* < 0.05.

### 2.4. Ethical Approval

This study has been approved by the Scientific Research Ethics Committee of Iuliu Hatieganu University of Medicine and Pharmacy Cluj-Napoca (No. 73/7.03.2024).

## 3. Results

### 3.1. Epidemiology and Cohorts Description

A total of 76 patients from Transylvania (Romania) were included in this study. The patients underwent 81 hospitalizations (episodes) for ORN or MRONJ, with an ORN to MRONJ ratio of 1:2.7. Out of 76 patients, 56 responded to the questionnaire. The patients were aged between 29 and 84 years old, with a mean ± standard deviation of 64 ± 11 years old (Table 1). Out of 76 patients, 71 patients were aged above 50 years (93.4%). Four patients were admitted to the hospital more than two times, with one female patient being hospitalized three times for both MRONJ and ORN. The male-to-female ratio was 1:1.3, and most of the patients had an urban place of living (81% patients). 

Alcohol and tobacco abuse was denied by most of the patients (92% consumed no alcohol and 85% consumed no tobacco). On one hand, out of the 54 patients suffering from tumors, 11 patients admitted alcohol and tobacco consumption, and on the other hand, out of the 54 patients treated with BPs, 3 admitted alcohol and tobacco consumption. The patients were ASA II-IV, with none of them being classified as ASA I (Table 1). Diabetes mellitus type 2 was found in 23 patients, and altered metabolic blood tests showing the presence of lipids (e.g., cholesterol) or proteins (e.g., albumin) were identified in 47 patients. Also, 29 patients had high inflammation on blood test parameters and 55 patients were immunosuppressed. 

The most common tumor localizations were in the head and neck region occurring in 26 out of 54 patients (48%), followed by 18 patients suffering from breast cancer (33%). The BP treatment for osteonecrosis caused MRONJ in 20 patients (Table 1). From the results of the responders of the questionnaire, we observed that the BPs were orally administered in osteonecrosis or tumor treatment in 17 cases (51% patients) and intravenous in 16 cases (48% patients), mostly in weekly (15 patients, 38%) or monthly (19 patients, 48%) regimens (Tabel 1). The treatment lasted for a mean ± standard deviation of 33 months ± 36 months with a minimum of 1 month, and a maximum of 168 months.

On one hand, using the AAOMS staging system for MRONJ, most of the patients were classified as Stage 2 (23 patients, 42.5%), followed by Stage 1 (10 patients, 18.5%), while 19 patients could not be classified (35,1%) due to the lack of data. On the other hand, ORN classified by Epstein et al.’s system identified most of the patients as Stage Ia (18 patients, 60%), followed by Stage IIa (8 patients, 26.6%), with no patients categorized as Stage IIb or IIIb. Also, two patients were non-responders to the questionnaire and could not be included in the staging.

The ORN was mostly related to head and neck cancers located in the oropharynx (two patients), nasopharynx (two patients), hypopharynx (two patients), tonsils (three patients), tongue (six patients), floor of the mouth (seven patients), retromolar space (one patient), and soft and hard palate (three patients). The treatment was performed by intensity-modulated radiation therapy (IMRT). IMRT was usually administered daily for 5 days, with 2 days’ break, for 4 or 5 weeks, depending on the oncological protocol, with a mean ± standard deviation of 4.68 ± 1.04 weeks. All the patients underwent external radiotherapy.

The most commonly affected bone was the mandible (48 patients), more specifically, the posterior mandible as observed in 40 patients (87% patients), with local and regional signs (Table 1). There were also some patients who did not experience any signs (18% patients). Among local signs, bone exposure, fistula, oroantral communication, and abscesses appeared in 36 patients (39.1%). Regional signs (42 patients (45.6%)) were mostly inflammation, maxillary sinusitis, and one case of facial palsy. There were also 14 patients with no obvious signs (15.2%). Patients complained about mild symptoms (pain only) in 22 cases, moderate symptoms (pain plus dysphagia and halitosis) in 32 cases, and severe symptoms associated with aggravated signs (fistula and oroantral communication) in 11 cases. 

### 3.2. Treatment Outcomes Evaluation 

A primary suture was used when the amount of gingiva permitted close suture (33 episodes, Table 1). On one hand, the PRF was used in 35 patients when no signs of active infection were identified, or when a significant amount of normal aspect bone remained. On the other hand, iodoform gauze packing was used in 13 patients when a large sequestrectomy with small remanent bone was present or when any sign of infection was present. Associated antibiotic therapy was administered using clindamycin (35 episodes) and aminopenicillins (Amoxicillin 32 episodes and Ampicillin 1 episode) as the antibiotics of choice. Second intention antibiotics, such as Cephalosporins (five cases), Piperacillin/Tazobactam (one patient), or the association between Beta-lactam, Gentamicin, and Metronidazole (seven patients), were in accordance with the antibiogram results. 

After this surgical and antibiotic treatment, most patients experienced reduced symptoms (45 patients (70.3%)). Also, the signs of healing were observed in most cases (47 patients (73.4%)). The PRF treatment reduced the symptoms and signs in 83% of cases compared to primary suture or iodoform gauze, which reduced by 65% and 71%, respectively (*p* > 0.05). The aminopenicillins and clindamycin reduced the signs and symptoms after antibiotic treatment in 28 out of 35 episodes (78.8%) and 25 out of 33 episodes (75.7%), respectively; the rest of the antibiotic regimen reduced the signs and symptoms in 7 out of 13 episodes (53.8%), compared to the first line of antibiotics. Also, there was no statistically significant difference between the clindamycin and aminopenicillin regimens’ treatment of bone necrosis regarding a reduction in the signs and symptoms (*p* > 0.05).

Firstly, PRF treatment was preferred equally for MRONJ and ORN, comparative to primary suture or iodoform gauze, which were mostly used for MRONJ. The PRF induced healing in 95.5% of ORN patients compared with 70% of MRONJ. 

Secondly, the treatment by primary suture and iodoform gauze was more effective in MRONJ compared to radiotherapy, with recurrence being more frequently seen after radiotherapy (25%) vs. Bis (0%).

Recurrence occurred in 18 patients (22.2%), the majority of them indicating the appearance of bone necrosis after 6 months. The recurrence of bone necrosis developed more often in those treated with primary suture or iodoform gauze (31%) compared to PRF treatment (17%) (*p* > 0.05), being identified after 6 months for most of the patients (9 of the 10 patients). Also, the recurrence was more frequent after PRF treatment for MRONJ rather than ORN (30% vs. 4.3%).

### 3.3. Factors That Interfere with the Treatment

Healing and recurrences differed between patients regarding various factors, including the different causes and associated treatment, age, ASA risk, and associated systemic pathologies.

Regardless of the type of treatment used, the rates of recurrence were almost similar for radiotherapy and BPs, with an earlier time of recurrence in patients treated with radiotherapy than in patients treated with BPs (4 < 6 months radiotherapy vs. 1 < 6 months BPs). However, the rates of recurrence for BPs depend on the route of administration. The oral administration of BPs showed less recurrence than parenteral administration (2 out of 23 vs. 8 out of 19). The symptoms and signs were more persistent in the parenteral administration of BPs (9 out of 19) compared to oral administration (1 out of 23, *p* = 0.003). Also, the duration of treatment influenced signs and symptoms. Patients with more than 1 year of BP administration complained about more intense symptomatology compared to patients with less than 1 year on BPs (17 out of 26 vs. 6 out of 12).

The tumors produced a more intense symptomatology compared with osteoporosis, with moderate or severe symptoms and regional signs occurring in 41 out of 53 tumor patients, compared to 6 patients out of 23 with moderate or severe symptoms and 9 patients out of 23 with regional signs. Also, head and neck tumor-related bone necrosis had a higher morbidity rate compared to other localizations (21 out of 26 patients with regional signs in head and neck tumors vs. 19 out of 28 patients with regional signs in other localizations) (*p* = 0.029).

The location of the jaw necrosis influenced the complaints. The posterior mandible had the most severe signs and symptoms (83.9% moderate and severe symptoms and 78% regional), followed by the posterior maxilla (44.4% moderate and severe symptoms and 38.9% regional signs). However, the postoperative signs and symptoms with persistency or aggravation were mostly seen in the mandible (13 out of 47 cases), with the posterior mandible having the greatest percentage (34.1%). The recurrence of necrosis was also seen more frequently in the mandible (16 out of 47), compared to the maxilla (4 out of 27), with the posterior mandible having the greatest percentage (34.1%).

Starting with the patients in their sixth decade of life, osteoporosis and tumors were prone to affect jaw bones and to produce MRONJ and ORN after each specific treatment. In those patients who had not yet reached their sixth decade of life, we report no osteoporosis associated with MRONJ and six patients who developed ORN. Most recurrences appeared in patients in their sixth and seventh decades of life, and most of the patients aged above 60 years old followed a BPs treatment for more than 1 year (26 out of 38 patients). 

On one hand, the symptoms and signs became more intense in ASA III patients, as 37 out of 59 presented with moderate or severe symptoms, and 44 out of 59 patients presented with regional signs compared to ASA II patients (6 out of 17). On the other hand, the ASA II patients presented healing in all cases, compared to ASA III and IV in which 44 out of 61 patients were healed (73.3%). 

The immunosuppression and blood test alteration induced changes in healing and recurrences. On one hand, immunosuppression was a negative predictor factor for healing, as 15 out of 54 (27.8%) patients reported having aggravation or a persistence of signs and symptoms after treatment, compared to only 1 out of 24 (4.2%) from non-immunosuppressed patients (*p* = 0.018). Also, recurrence occurred more often in immunosuppressed patients (29.6%) compared to non-immunosuppressed patients (4.2%) (*p* = 0.026). On the other hand, patients with elevated inflammatory blood tests (e.g., C-reactive protein) complained about more intense signs and symptoms (10 out of 27, 40.7%) compared to patients with normal inflammatory blood tests (7 out of 36, 19.4%; *p* = 0.204). Also, patients with an intense inflammatory status associated with bone necrosis had difficulty in healing properly (16 out of 27 patients, 67% healing) compared to the ones with a reduced inflammatory status (29 out of 36, 80.6%; *p* = 0.336). The time of recurrence was mostly less than 6 months in the intense inflammatory status patients. We observed that the aminopenicillins were the preferred regimen in the intense inflammatory status patients (46.9%) compared to clindamycin (37.5%) (*p* = 0.598). Additionally, in the immunosuppressed group aminopenicillins were preferred (51.3%) over clindamycin (34.8%), compared to non-immunosuppressed patients where clindamycin was preferred (62.1% clindamycin vs. 31% aminopenicillins, *p* = 0.024). 

We identified no difference in healing in diabetes mellitus patients (69.9%, 15 out of 22 patients) compared to non-diabetes mellitus patients (77.5%, 30 out of 40 patients; *p* = 0.590). However, the recurrence of bone necrosis appeared more frequently in diabetes mellitus patients compared to non-diabetes mellitus patients (34.8% vs. 22.5%, *p* = 0.445). In diabetes mellitus patients, clindamycin was more frequently used (55.6%,15 out of 33 patients) compared to aminopenicillins (29.6%, 8 out of 35 patients), while in non-diabetic patients aminopenicillin was preferred (52.1%) compared to clindamycin (37.5%, *p* = 0.204).

The patients who smoke and consume alcohol had more severe signs, 77.8% of them having regional symptoms and 61% moderate symptoms, compared to non-smokers and non-drinkers patients (59% regional signs and 41% moderate signs). We also observed an increase of 10% in the recurrence rate in patients who were smoking and abusing alcohol, compared to patients who did not. No statistical significance was observed.

## 4. Discussion

To our knowledge, this is the first study that reports the epidemiology of head and neck MRONJ and ORN in a national healthcare institution in Romania, as well as the outcomes of surgically treated patients with MRONJ and ORN.

The data in the literature are discordant when it comes to the quantification of MRONJ or ORN prevalence, depending on treatment centers, number of patients, or the location of the bone lesions [8]. Prevalence is usually higher for head and neck cancer patients [16,17]. In our study, we observed a larger number of tumor patients compared to osteoporosis patients. ORN prevalence is estimated between 0.7% and 15%, whereas MRONJ prevalence ranged from 0.043% for oral BPs to 1.03% for intravenous BPs [19,20]. Compared to these findings where the ORN prevalence is 6 to 15 folds higher than MRONJ, in our study we identified a prevalence for MRONJ that is almost 3 folds higher. 

Out of all the tumor sites, head and neck malignancies treated by radiotherapy were the most common cause of ORN in our study. Even if IMRT has the benefit of concentrating the radiation beam around the tumor, minimizing the damage of the surrounding healthy tissues, it has a direct impact on the jaw [21]. The breast cancer treatment using BPs had a major impact, as it has a high risk of bone metastases, with this pathology being almost as likely as osteoporosis to cause MRONJ [22]. In our study, the breast was the most common tumor site, with an incidence rate of 28.5%. 

Higher doses of BPs, their intravenous route of administration, and their long-term administration have been associated with a lower rate of postoperative healing [23]. A higher prevalence of MRONJ was seen for intravenous BPs rather than for oral BPs (1.03% compared to 0.043%) [19,24]. In our study, there were almost equal numbers of patients treated with oral BPs and intravenous BPs, but with higher rates of persistent symptoms and recurrences after intravenous BPs.

The degree of bone exposure and severe signs such as fistula or oro-antral and oro-nasal communications can take more time to heal as they are serious complications [25]. We identified that 16.9% of cases had severe complications. The most common site of MRONJ is the mandible, having a worse prognosis [26]. In our study, the mandible was the most common site in 59% of cases, with a 49.3% incidence rate for the posterior mandible. The posterior mandible necrosis was associated with more intense discomfort for the patients and higher recurrence rates compared to other localizations.

Oncological patients are difficult to manage due to their immunosuppressed status and medical history, as they are more prone to a range of complications [27]. In our study, all patients with tumor-related bone necroses were immunosuppressed. Different from other studies, we identified no ASA I patients [28]. This could be explained by the variety of medical healthcare problems that these patients had, and by age, with most patients being over 50 years old. High-ASA risk patients were correlated with a higher risk of postoperative complications, and low ASA risks patients, such as ASA I and II, were correlated with being more able to undergo successful surgical treatment [29,30,31,32].

On one hand, diabetes is a risk factor for osteonecrosis of the jaw due to reduced osteoclastic differentiation and activity, and this is linked to a poor prognosis [33,34,35,36,37,38]. However, similar to other research that showed no correlation between bone necrosis, healing, and diabetes, we found no statistically significant correlation of healing with reference to diabetes [36,37,38]. 

On the other hand, immunosuppression and elevated inflammation markers can be associated with a higher risk of incomplete healing, interacting with the treatment outcome [39,40,41,42]. Additionally, we identified that immunosuppressed patients had a higher recurrence rate, with a persistence of signs and symptoms. Also, we observed that patients with high inflammation parameters had healing difficulties but with no statistical significance.

Surgical treatment has been shown to have better results than conservative methods [23]. Studies show that PRF treatment reduces symptoms visibly postoperatively after a few days, as well reducing signs and improves bone healing significantly on follow-up evaluations [43]. In MRONJ patients, postoperative evaluations showed fast soft tissue healing, no bone exposure, no signs of infection, and no complications [44]. In ORN treatment, PRF has been proven to be beneficial as well [45]. Iodoform gauze packing is a well-used antiseptic and germicide that is applied in various treatment protocols, including MRONJ and ORN, preventing infections and sustaining healing [46]. High healing rates have been discussed for primary suture if there is an appropriate surgical resection, with low rates of recurrence observed for both MRONJ and ORN [47]. Different from other research, in our study, PRF improved healing and reduced recurrence rates compared to primary suture and iodoform gauze packing, and had high rates of reduced symptoms [48,49]. However, more studies are needed for a proper conclusion. PRF seems to be equally effective when it comes to radiotherapy or BPs. Some studies show that while all patients with radiotherapy treated with PRF showed signs of complete healing, almost all patients with BPs treated with PRF healed completely [7]. Additionally, some studies show that almost all patients (17 out of 19) treated with primary suture healed completely [50]. In our study, radiotherapy patients treated with PRF had a better outcome than BPs-treated patients. In opposition, radiotherapy patients treated with primary suture experienced higher rates of recurrence compared to patients treated with BPs. 

When it comes to vices, we did not obtain any positive correlation between smoking or drinking and treatment outcome or recurrence. This might be because patients have a tendency to hide their vices, due to stigmatization of these habits, making it a taboo subject [51,52]. Although, many patients quit smoking or drinking after being diagnosed with cancer, realizing that it might be beneficial [53,54].

Limitations of this study may be considered when interpreting the results, such as the low number of patients, especially as not all patients respected the follow-up protocol and answered the questionnaire. Additionally, we did not evaluate or consider the patients’ oral hygiene status, which may influence healing. We did not include any patients treated with Denosumab in our study. The bisphosphonate treatment was conducted by the endocrinologist or oncologist; therefore, we did not prescribe any drug holiday, but we treated the effects of the general treatment. However, this research is the first Romanian study that presents and evaluates a treatment protocol for MRONJ and ORN, making it a potential starting point for future prospective studies.

## 5. Conclusions

MRONJ and ORN patients are challenging to treat. A protocol that associates necrotomy, bone planning, antiseptic lavage, PRF, and antibiotic administration helps reduce signs and symptoms. PRF treatment proved to be a reliable method to help heal the necrotic bone sites for both ORN and MRONJ patients. Also, The high-ASA risk patients and immunosuppressed patients are more prone to recurrence and persistent signs and symptoms. Intravenous BPs produce more intense symptomatology compared to oral administration. The posterior mandible is more difficult to treat compared to other sites.

## Figures and Tables

**Table 1:** Patients’ demographic and clinical characteristics.

Selected Parameters (n = Patients (%))		
Sex	F	44 (57.9%)
	M	32 (42.1%)
Living place	U	63 (82.9%)
	R	13 (17.1%)
Cause	Tumor	54 (71%)
	Osteoporosis	20 (26.3%)
	Tumor and osteoporosis	2 (2.6%)
Alcohol and tobacco abuse *	Cigarettes	8 (10.5%)
	Alcohol	3 (3.9%)
	Cigarettes and alcohol	3 (3.9%)
Tumor location (systems)	Head and neck	26 (48%)
	Blood and lymphatic system	4 (7.4%)
	Gastrointestinal	2 (3.7%)
	Breast and genitals	18 (33.3%)
	Internal organs	2 (3.7%)
Treatment type	Bisphosphonates	46 (60.5%)
	Radiotherapy	22 (29%)
	Bisphosphonates and radiotherapy	8 (10.5%)
Route of administration for bisphosphonates *	Intravenous	16 (48.48%)
	Oral	17 (51.51%)
Frequency for bisphosphonates *	Monthly	19 (48.7%)
	Weekly	15 (38.4%)
	Daily	5 (12.8%)
Period interval for bisphosphonates *	≤3 months	2 (7.6%)
	4–6 months	4 (15.3%)
	7 months–1 year	2 (7.6%)
	1–5 years	15 (57.7%)
	>5 years	3 (11.5%)
ASA risk	I	0
	II	17 (22.3%)
	III	53 (69.7%)
	IV	6 (7.8%)
Localization ^+^	Anterior maxilla	12 (14.8%)
	Posterior maxilla	20 (24.6%)
	Anterior mandible	6 (7.4%)
	Posterior mandible	40 (49.3%)
	Anterior and posterior maxilla	1 (1.2%)
	Anterior and posterior mandible	2 (2.4%)
Signs *	None	14 (15.2%)
	Local	36 (39.1%)
	Regional	42 (45.6%)
Symptoms *	Mild	22 (33.8%)
	Moderate	32 (49.2%)
	Severe	11 (16.9%)
MRONJ sites Stage (AAOMS)	1	10 (18.5%)
	2	23 (42.5%)
	3	2 (3.7%)
	Could not be classified	19 (35.1%)
ORN sites Stage (Epstein et al.) [17]	Ia	17 (60%)
	Ib	1 (3.3%)
	IIa	7 (26.6%)
	IIb	0
	IIIa	1 (3.3%)
	IIIb	0
	Could not be classified	2 (6.6%)
Surgical treatment ^+^	Primary suture	33 (40.7%)
	Iodoform gauze	13 (16%)
	PRF	35 (43.2%)
After treatment signs *	Healing	47 (73.4%)
	Persistence	14 (21.8%)
	Aggravated	3 (4.6%)
After treatment symptoms *	Reduced	45 (70.3%)
	Persistence	18 (28.1%)
	Aggravated	1 (1.5%)
Antibiotherapy ^+^	Clindamycin	35 (43.2%)
	Aminopenicillins	33 (40.7%)
	Second intention antibiotics	13 (16.1%)
Recurrence ^+^	Yes	18 (22.2%)
	No	63 (88.8%)
Time of recurrence (months)	1–6	6 (33.3%)
	>6	12 (66.6%)

* responders to the questionnaire, ^+^ episodes of bone necrosis, F = female, M = male, U = urban, and R = rural.

## Data Availability

The data presented in this study are available on request from the corresponding author due to data confidentiality of patients.
